# A Curcumin Analog Exhibits Multiple Biologic Effects on the Pathogenesis of Alzheimer’s Disease and Improves Behavior, Inflammation, and β-Amyloid Accumulation in a Mouse Model

**DOI:** 10.3390/ijms21155459

**Published:** 2020-07-30

**Authors:** Ih-Jen Su, Hong-Yi Chang, Hui-Chen Wang, Kuen-Jer Tsai

**Affiliations:** 1 Department of Biotechnology and Food Technology, Southern Taiwan University of Science and Technology, Tainan 71005, Taiwan; czeus1974@gmail.com; 2 Merry Life Biomedical Company, Tainan 71005, Taiwan; sabrinawang@tmlbio.com; 3 Institute of Clinical Medicine, College of Medicine, National Cheng Kung University, Tainan 704, Taiwan; 4 Research Center of Clinical Medicine, National Cheng Kung University Hospital, College of Medicine, National Cheng Kung University, Tainan 704, Taiwan

**Keywords:** Alzheimer’s disease therapy, curcumin analog, TML-6, aging, bioavailability

## Abstract

Drugs for the treatment of Alzheimer’s disease (AD) are in urgent demand due to the unmet need and the social burden associated with the disease. Curcumin has been historically considered as a beneficial product for anti-aging and AD. However, many efforts to develop curcumin for clinical use are hindered mainly due to its poor bioavailability. Recent development in drug delivery and structural design has resolved these issues. In this study, we identified a small molecule, TML-6, as a potential drug candidate for AD through screening a panel of curcumin derivatives using six biomarker platforms related to aging biology and AD pathogenesis. The structural modification of TML-6 is designed to improve the stability and metabolism of curcumin. Cell biological studies demonstrated that TML-6 could inhibit the synthesis of the β-amyloid precursor protein and β-amyloid (Aβ), upregulate Apo E, suppress NF-κB and mTOR, and increase the activity of the anti-oxidative *Nrf2* gene. In the 3x-Tg AD animal model, TML-6 treatment resulted in significant improvement in learning, suppression of the microglial activation marker Iba-1, and reduction in Aβ in the brain. Although TML-6 exhibited a greater improvement in bioavailability as compared to curcumin, formulation optimization and toxicological studies are under development to assure its druggability. Taken together, TML-6 meets the current strategy to develop therapeutics for AD, targeting the combination of the Aβ cascade and aging-related biology processes.

## 1. Introduction

Alzheimer’s disease (AD) is the most common cause of dementia. It has been estimated that the total number of AD individuals will increase to approximately 74.7 million by 2030 and the cost of healthcare will increase to USD 1 trillion annually by 2050 [1]. More than hundreds of clinical trials have been conducted in the AD field in the past decades [2], however, only a few symptom-relief agents reach the markets. This is mainly due to the inadequate understanding of the complex pathogenesis of AD, the inappropriate design of clinical trials, and the lack of reliable diagnostic tests or biomarkers for patient inclusion [3]. Thus, there exists an urgent demand for developing therapeutic drugs for AD patients to delay and arrest the disease progression, or even to reverse the cognitive function decline, especially for patients at the non-demented or early stage disease. 

The dominant AD pathogenesis theory is the accumulation of Aβ and Tau proteins in the patient’s brain [4]. The senile plaques in AD are composed of Aβ fibrillary proteins which promote Tau hyper-phosphorylation, neuron cell death, cognitive dysfunction and finally disruption of normal brain function [5,6,7]. The Aβ peptide is composed of 40–42 amino acids and generated by proteolytic processing of the larger amyloid precursor protein (APP), which produces Aβ by the cleavage of paired enzymes β-secretase and γ-secretase [8]. Therefore, the drug development initiatives in the past decades have mainly targeted Aβ, tau, and the secretase [2,9]. Unluckily, the majority of clinical trials in the past decade were unsuccessful, many of which failed or withdrew in the late stage trials [2,3,10]. Most recently, the clinical trials using anti-Aβ monoclonal antibodies to prevent Aβ aggregation or degrade Aβ aggregates resulted in disappointing outcomes [3,11]. In May 2018, the US National Institutes of Health organized an expert summit to discuss the strategy to develop preventive and therapeutic measures for AD control, and recommended that novel mechanistic insight and precision medicine must be enabled to clearly understand the multifaceted pathogenesis of AD, and to develop drugs targeting at combining the Aβ cascade and other disease-modifying pathways of AD [12].

Besides the dominant APP/Aβ/tau hypothesis, several alternate mechanisms or disease-modifying pathways of AD pathogenesis have been proposed. Among them, apo-lipoprotein E (Apo E) represents one important factor in AD pathogenesis. ApoE plays a major role in cholesterol biology and is also involved in the catabolism of Aβ clearance [13]. Genetic studies have indicated that the polymorphism of the ApoE isoform ε4 is a greater risk factor in comparison with isoforms ε2 and ε3 for late-onset AD [14]. Although several mechanisms associated with ApoE-related AD have been reported, the exact role of ApoE in AD pathogenesis remains to be clarified [14]. In AD patients, the ApoE levels in plasma tend to be lower than healthy individuals as observed in cohort and meta-analysis studies [15,16]. Therefore, targeting at ApoE in all *APOE* genotypes may provide a strategy for the treatment of AD.

Lately, neuro-inflammation and the autophagy machinery dysfunction are among the most notable AD pathogenesis. Neuro-inflammation associated with Aβ accumulation and microglial activation has been found to play a critical role in AD progression [17,18]. Patients with rheumatoid arthritis receiving long-term treatment of nonsteroidal anti-inflammatory drugs showed protective effects on AD and had an approximately 50% reduction in the risk of AD development [19]. Dysfunction of the autophagy machinery and the activation of anti-aging mTOR has been reported to cause the enhancement of Aβ production and deposition through APP proteolysis by upregulating β- and γ-secretases [20]. Therefore, the suppression of mTOR phosphorylation may provide a therapeutic strategy for AD.

The most provocative theory related to AD pathogenesis recently proposed is the role of oxidative stress and free radicals associated with the aging process [21,22]. The production of free radicals during oxidative stress could result in the impairment of DNA repair and cause protein mutations and lipid peroxidation, which is strongly implicated in aging-related diseases and AD [22]. The activation of the Nrf2-mediated antioxidant system has been proposed as an important strategy to downregulate oxidative stress and is also related to the pathogenesis in AD [23]. Antioxidant or anti-NrF2 medication at the early stages of the disease may hence provide prevention of AD. Therefore, efforts to develop a drug with multifaceted functions or to combine drugs that target the combination of the Aβ cascade and aging-related biologic pathways will constitute the novel strategy to develop drugs for AD treatment.

Herbs and natural compounds have been found to exhibit diverse biological and medical functions throughout the medical history [24,25]. Curcumin is among one of the most attractive compounds that serve as a candidate for anti-aging and AD therapy, owing to the low prevalence of AD in India where curcumin is included in the daily diet [25,26]. Curcumin has been effective against a wide variety of aging-related diseases through multiple molecular mechanisms [26,27,28,29,30]. Several studies have indicated that curcumin has neuroprotective and cognitive-improving potential which could prevent AD and other neurodegenerative diseases [31,32]. Despite ample scientific reports supporting the biological functions of curcumin in human diseases, a huge controversy exists regarding curcumin’s capability as a drug, mainly due to the broad or nonspecific interaction with target molecules [33,34] and its poor bioavailability [35]. Nevertheless, the recent development of formulation technology and structural modification of curcumin have solved this problem. The most recent progress of curcumin in AD clinical trial is a double-blind, placebo-controlled 18-month trial conducted by Gary Small and his group at University of California, Los Angeles (UCLA), which showed therapeutic effects on memory and reduction in brain amyloid in non-demented adults by the administration of a bioavailable form of curcumin [36]. This report provides the first proof of concept in the development of curcumin for AD prevention and therapy using the current AD biomarkers.

In this study, we identified TML-6 as a potential AD drug candidate based on the evidence including: (I) cell biology characterization through screening a library of curcumin analogs using biomarker technology, which includes the key factors related to aging biology and AD pathogenesis as mentioned above; (II) using a triplex transgenic AD mouse model to verify the in vitro biologic effects of TML-6; (III) pharmacokinetic studies of TML-6 to evaluate the feasibility of TML-6 for future drug development. TML-6 was found to effectively inhibit multiple pathways of AD pathogenesis, including the inhibition of the synthesis of APP and Aβ, the upregulation of levels of ApoE, the suppression of the inflammation-related phosphorylated NF-κB and the aging-related mTOR, and the increase in the expression of the anti-oxidative stress gene Nrf2. In the triplex AD transgenic animal study, TML-6 could improve the memory behavior in the Morris water maze test, suppress microglial activation, and reduce the brain levels of Aβ. TML-6 displayed greatly improved pharmacokinetic readouts as compared to traditional curcumin. In summary, we propose TML-6 as a potential AD drug candidate which meets the current strategy to develop drugs targeting the combination of the Aβ cascade and aging-related biologic pathways for AD. All the cell biology studies, pharmacokinetic studies, and AD transgenic mouse studies were conducted using unformulated TML-6. Formulation optimization is under development before the preclinical toxicology is conducted.

## 2. Results

### 2.1. Structural and Physico-Chemical Characterization of TML-6

**Figure 1:**
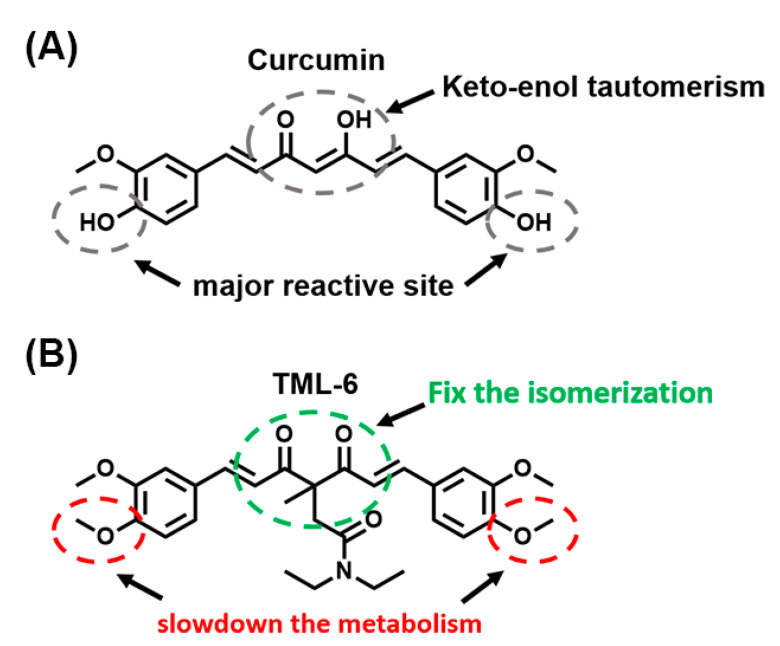
Comparison of traditional curcumin and TML-6 through its chemical structure. (**A**) The medical use of traditional curcumin is limited by its water insolubility (hydrophobic), low intestinal absorption, rapid metabolism, which leads to poor bioavailability. (**B**) TML-6 modified from traditional curcumin conserves the bioactivity of curcumin, becomes more stable, and slows down in metabolism. However, preliminary studies revealed that both curcumin and TML-6 had poor water solubility and need to be optimized by formulation.

As compared to the orange to yellow color of curcumin, TML-6 has a light yellowish to white color. A comparison of the chemical structures of curcumin and TML-6 is shown (Figure 1A,B), respectively. The two hydroxyl groups on curcumin’s benzene structure are hot spots for β-glucuronidation and are replaced by methoxyl groups in TML-6 to slow down the metabolism. Furthermore, the structure of TML-6 has fixed the isomerization which is more stable in acidic pH than curcumin. Therefore, by structure design and prediction analysis, TML-6 will slow down the metabolism and become more stable under the acidic or basic condition than curcumin. In the beginning, a total of twelve curcumin derivatives were conducted to screen their therapeutic potential. Based on chemical prediction and cell biological characterization, two curcumin analogs remained. TML-6 was finally chosen as a candidate because the other one had high cytotoxicity.

In preliminary studies, the water solubility of curcumin and TML-6 was less than 0.1 mg/mL for both. TML-6 exhibited a higher degree of lipophilicity, which makes it a feature of poor water solubility. In brain penetration studies, TML-6 fed at an oral dose of 150 mg/kg could be detected at a level of 3 ng/mL (10% of the plasma pharmacokinetics (pK) levels) in the AD transgenic mouse brain. However, TML-6 could not be detected in the normal mouse brain at the same dose. The data are interesting and raise the possibility of the dysfunction or leakage of blood–brain barrier (BBB) in AD mice. We are designing a study to clarify the BBB penetration after feeding TML-6 to normal and AD mice at different ages.

To evaluate whether TML-6 can be developed as a therapeutic drug for AD, the following three categories of studies were further conducted: (I) cell biology of aging and AD-related biomarker studies; (II) the efficacy of TML-6 in the triplex AD transgenic mouse model; and (III) pharmacokinetic studies of curcumin and TML-6. The results are shown in the following sections.

### 2.2. Cell Biologic Characterization of TML-6

#### 2.2.1. Cytotoxicity of TML-6 Examined by Cell Counting Kit-8 (CCK-8)

**Figure 2:**
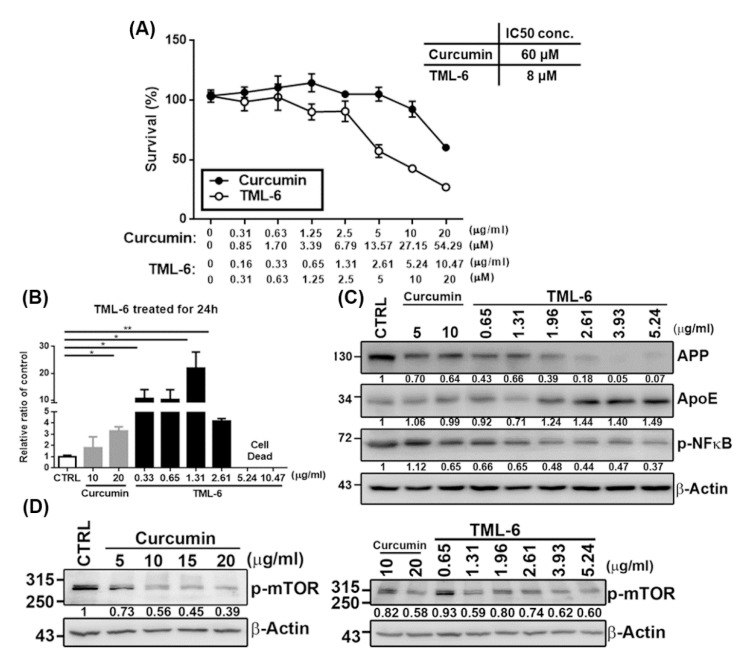
The cytotoxicity studies, inhibitory concentration (IC)50, and biologic studies of TML-6. TML-6 could transcriptionally activate the Nrf2 promoter, suppress the expression of the amyloid precursor protein (APP) and phospho-NF-κB, and in contrast, increases the ApoE and phospho-mTOR expression in Huh-7 cell line. (**A**) The cytotoxicity (IC50 assay) of traditional curcumin and TML-6 were examined by CCK-8 in Huh-7 cells for 24 h. The working concentration of traditional curcumin and TML-6 was expressed in μM and μg/mL separately for comparison. The IC50 of curcumin and TML-6, 60 and 8 μM, respectively, is expressed at the right side of Figure 2A. In the following studies, the dose concentration was expressed in μg/mL only, as indicated in Figure 2B–D. (**B**) The luciferase plasmid contains the Nrf2 promoter co-transfected with pRL-TK (Renilla) into Huh-7 cells by Lipofectamine 2000. Transfected cells were treated with curcumin and TML-6 for 24 h, and the Nrf2 promoter activity was analyzed by the Dual-Luciferase Report assay system. TML-6 could exhibit transcriptional activation of the *Nrf2* gene in a dose-dependent manner, with the highest activity at a concentration of 1.32 µg/mL, which was 12.1- and 6.6-fold stronger than the traditional curcumin at a dose of 10 and 20 µg/mL, respectively. Data represent the mean with a standard deviation (SD) error bar and *p* value < 0.05 was considered significant (* *p* < 0.05, ** *p* < 0.01). The Nrf2 promoter plasmid revealed a higher cytotoxicity beyond 5.24 μg/mL. (**C**) Huh-7 cells were treated with different doses of traditional curcumin and TML-6 for 24 h. Western blotting was performed to examine Alzheimer’s-related protein molecules such as APP, Apo E, inflammatory marker-phosphorylated NF-κB (**C**), and phosphorylated mTOR (**D**). The quantification of protein expression levels in APP, ApoE, p-NFkB and p-mTOR were normalized by β-Actin and are shown as fold induction in comparison to the untreated control (CTRL) under immunoblotting data in each panel. These AD biomarker proteins revealed a dose-dependent manner in response to TML-6 treatment.

The cell viability of TML-6 was examined by CCK-8 in Huh-7 cell line for 24 h incubation and the working concentration was indicated (Figure 2A). The data from the CCK-8 assay showed that TML-6 revealed no cytotoxicity in Huh-7 cells at concentrations below 5 μM (equivalent to 2.61 µg/mL). The concentration was expressed in molarity and ng/mL, as indicated (Figure 2A). For practical use in drug development, the concentration was expressed in ng/mL in the following studies. The half maximal inhibitory concentration (IC50) value of TML-6 in Huh-7 cells at 4.19 µg/mL (8 μM). However, there was no obvious cytotoxicity of traditional curcumin, despite the highest concentration of 20 µg/mL used in this study (Figure 2A).

#### 2.2.2. TML-6 Could Ameliorate the Antioxidative and Anti-Aging Effect through Transcriptional Activation of the *Nrf2* Gene

The activation of the Nrf2-mediated antioxidant system has been proposed as an important strategy to downregulate oxidative stress and is also related to the pathogenesis of anti-aging and AD [23]. To verify whether TML-6 could exhibit antioxidant activity, we studied the transcriptional activity of the *Nrf2* gene promoter after treating with TML-6, using the luciferase reporter assay. Our data showed that TML-6 could exhibit transcriptional activation of the *Nrf2* gene in a dose-dependent manner, with the highest activity at a concentration of 1.32 µg/mL, which was 12.1- and 6.6-fold stronger than the traditional curcumin at a dose of 10 and 20 µg/mL, respectively (Figure 2B). As shown in Figure 2B, the cytotoxicity was found at a higher concentration (beyond 5.24 µg/mL) of TML-6 when transiently transected with the Nrf2 promoter into Huh-7 cells.

#### 2.2.3. TML-6 Exhibited Multiple Biologic Effects on AD-Related Biomarkers in Protein Levels, including APP (Amyloid Precursor Protein), Apo E, and Phospho-NF-κB

To examine the protein expression levels of AD-related biomarkers, Huh-7 cells were treated with traditional curcumin and TML-6 for 24 h, followed by Western blotting analysis. Our result indicated that the protein expression levels of APP and phospho-NF-κB exhibited a dose-dependent reduction after treating with TML-6 for 24 h. TML-6 reduced the APP protein expression level by 60% at a dose of 1.96 µg/mL after 24 h treatment, whereas traditional curcumin reduced the APP level by 30% at a dose of 5 µg/mL compared with the medium control (Figure 2C). TML-6 also decreased the level of phosphorylated NF-κB by about 50% at a dose of 1.96 µg/mL after 24 h treatment, while traditional curcumin decreased the level of phosphorylated NF-κB by 35% at a dose of 10 µg/mL compared with the medium control (Figure 2C). On the other hand, the protein expression levels of ApoE were significantly increased after TML-6 treatment. The Western blotting data indicated that TML-6 could induce the protein expression level of ApoE by approximately 44% at a dose of 2.62 µg/mL compared to both the medium and curcumin control (Figure 2C).

#### 2.2.4. TML-6 Inhibits the mTOR Signaling Pathway through the Suppression of Phospho-mTOR

Recent studies reported that mTOR signaling pathway links to the aging-dependent cognitive decline and acts as a critical effector of cerebrovascular dysfunction in AD [37,38]. On the other hand, the activation of mTOR enhances Aβ production and also leads to autophagy dysfunction in the aging process, which results in the disruption of Aβ clearance and increases Aβ deposition [39]. Therefore, the inhibition of phospho-mTOR may not only extend life span but also provide a therapeutic strategy against AD. In this study, Huh-7 cells were treated with traditional curcumin and TML-6 for 24 h followed by Western blotting analysis to examine mTOR activation. The data showed that phospho-mTOR was suppressed in a dose-dependent manner when Huh-7 cells were treated with traditional curcumin (Figure 2D-left panel). To examine the phospho-mTOR suppression by TML-6, we used traditional curcumin as a control at concentrations of 10 and 20 µg/mL. Our data showed that phospho-mTOR was suppressed in all the concentrations of TML-6-treated Huh 7 cells. We also found that the average of phospho-mTOR reduced by about 30% when the dose of TML-6 was below IC50 (between 0.65~3.93 µg/mL) after treating with TML-6 (Figure 2D-right panel). Results from quantification also indicated that TML-6 reduced the same level of phospho-mTOR at a concentration of 5.24 µg/mL, as compared to curcumin at 20 µg/mL, with about 4-fold potency of activities. These results indicate that the inhibition of phospho-mTOR by TML-6 is more effective than traditional curcumin (Figure 2D).

#### 2.2.5. TML-6 Suppresses Aβ Production in N2a/APPswe Stable Cells

**Figure 3:**
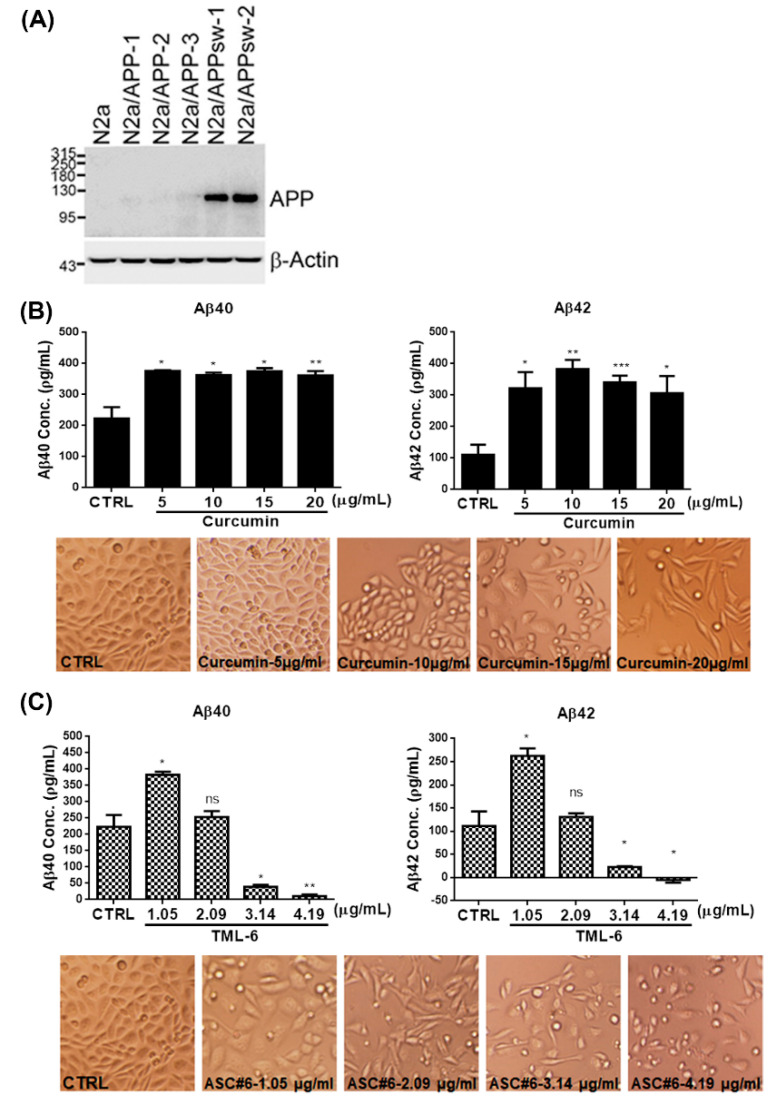
TML-6 suppresses Aβ production in the N2a/APPswe stable cell line. (**A**) The N2a cell line showed stable over-expression of APP with wild-type full-length (N2a/APP) and Swedish mutant (N2a/APPswe) cells by Western blotting. The N2a/APPswe stable cell line was used to examine Aβ 40 and Aβ42 levels in culture medium in response to traditional curcumin and TML-6 by ELISA test. N2a/APPswe stable cells were treated with different doses of traditional curcumin (**B**) and TML-6 (**C**) as indicated in the figures. The cell morphological change or viability in each treatment was captured and represented for comparison at the lower panel, as indicated. After 24 h incubation, the culture medium was examined for levels of Aβ40 and Aβ42 by LEGEND MAX™ β-Amyloid X-40 and X-42 ELISA Kit. The significant differences between the treated and control cells are represented as the mean with a standard deviation (SD) error bar, and *p* value < 0.05 was considered significant (* *p* < 0.05, ** *p* < 0.01, *** *p* < 0.001). The cell density, viability, and morphological changes by micrographs were similar between curcumin and TML-6, probably due to cytotoxicity at the highest doses of curcumin (20 μg/mL) and TML-6 (4.19 μg/mL) in N2a/APPswe stable cells.

Previous studies have demonstrated that curcumin can inhibit Aβ oligomerization and decreases amyloid [40]. In this study, the mouse neuroblastoma cell line N2a which harbors stable overexpression of APP with wild-type (WT) was used, and Swedish mutations were designed as N2a/APP and N2a/APPswe stable cell lines, respectively. The protein expression of APP was confirmed by Western blotting. Since the full-length WT APP plasmid has kicked out in N2a/APP stable cells, we used N2a/APPswe stable cell line for further study (Figure 3A). To examine whether the Aβ production was affected by TML-6, the cell line N2a/APPswe was treated with traditional curcumin and TML-6 for 24 h and kept the supernatant for ELISA analysis. The production of Aβ levels was examined in culture medium by using Aβ40 and Aβ42 ELISA kit. Our data showed that TML-6 reduced the production of Aβ40 and Aβ42 between 1.05, 2.09 and 3.14 μg/mL (equal to 2, 4 and 6 μM) in a dose-dependent manner. The least levels of Aβ40 and Aβ42 were reduced to approximately 86% and 80% at a concentration of 3.14 µg/mL of TML-6 as compared with the control group (Figure 3C). However, the levels of Aβ40 and Aβ42 after treating with traditional curcumin unexpectedly increased to levels higher than those of the control (CTRL) throughout the treatment at different doses, even cytotoxicity at the highest concentration of 20 µg/mL was demonstrated (Figure 3B). This phenomenon was also observed for low-dose (1.05 μg/mL) TML-6 treatment; however, the Aβ40 and Aβ42 levels started to decrease at a TML-6 dose of 2.09 μg/mL. This finding was supported by a previous observation that low-dose of curcumin can stimulate cell proliferation and potentially lead to increasing Aβ production in the N2A/APPswe stable cell line [41,42]. Therefore, our data indicate that TML-6 can significantly suppress the production of Aβ40 and Aβ42 in N2a/APPswe cells.

### 2.3. TML-6 Improved the Learning Behaviors, Significantly Suppressed the Aβ Levels and Iba-1 Expression in the Brain of 3xTg AD Transgenic Mice

To verify the efficacy of TML-6 as a potential therapeutic for AD, we conducted the animal study by using six-month-old 3xTg (mutations: APP_KM670/671NL_, MAPT_P301L_ and PSEN1_M146V_) AD transgenic mice fed with normal diets (vehicle), diets mixed with traditional curcumin, or diet containing TML-6 (150 mg/kg/day) for four months. After 4 months, the 3xTg AD mice were examined for learning and memory behavior by the Morris water maze test. The brain tissues were verified for the expression of Aβ levels and the microglial activation marker, Iba-1, by immunofluorescence staining.

**Figure 4:**
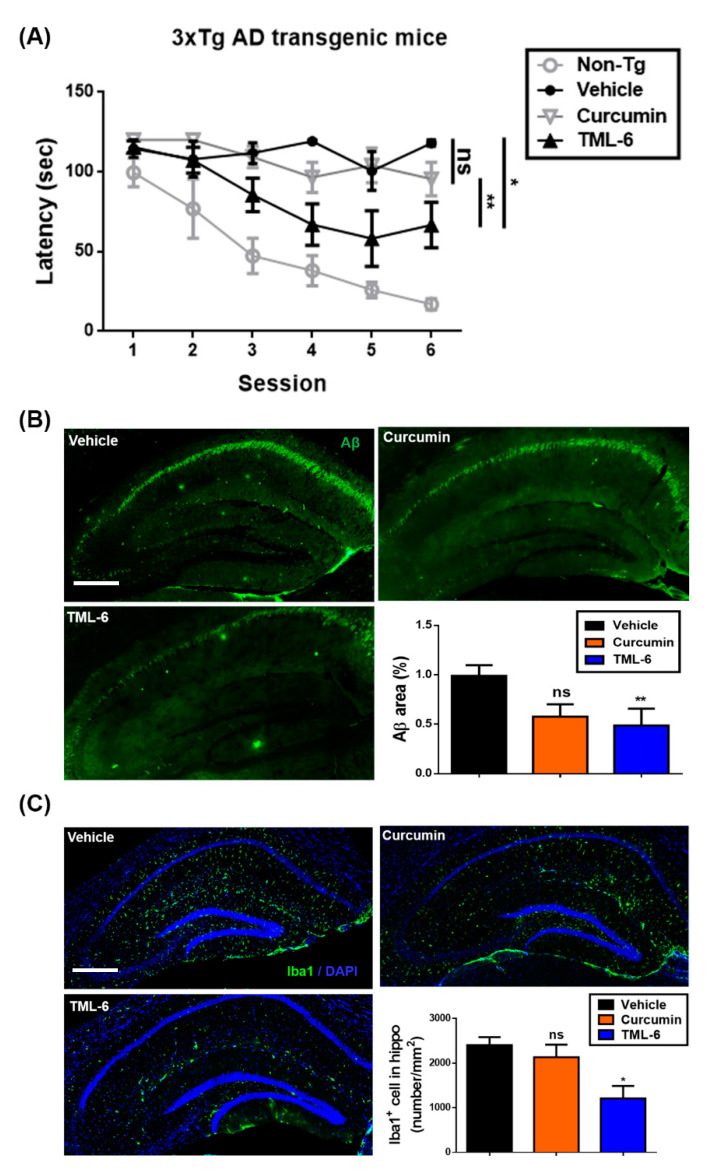
TML-6 improved the behavior test and reduced the brain levels of Aβ and the inflammatory biomarker Iba1 in the 3x-Tg AD mouse model. (**A**) Six-month old 3x-Tg AD mice were fed with normal diets (vehicle), or diets containing traditional curcumin and TML-6 for four months at a dosage of 150 mg/kg (each group contained five mice, *n* = 5). After four months of treatment on the experimental diet, animals were subjected to a behavior test in the form of the Morris water maze. The figure shows data from vehicle, curcumin and TML-6 fed different chow diets, with triplex-Tg AD mice and non-transgenic mice (Non-Tg) serving as the control. (**B**) The Aβ levels in mouse brains in 3x-Tg AD mice were examined at the end of behavior test after four-month feeding with the treatment of normal diets, traditional curcumin and TML-6. Mouse brain sections were examined by immunofluorescence staining. The immunofluorescence staining of Aβ was quantified and is represented in the panel. Scale bar: 200 μm. (**C**) Mouse brain sections were also examined for the inflammatory biomarker Iba1 by immunofluorescence staining. The images of Iba-1 levels were represented and quantified as indicated in the panel. The significance of the behavior test, Aβ, and Iba-1 levels between the mice treated with normal diets (vehicle) and either curcumin or TML-6 was determined by paired *t*-test. Asterisks denote data representing the mean with a standard deviation (SD) error bar, and *p* value < 0.05 was considered significant (* *p* < 0.05 and ** *p* < 0.01).

In the Morris water maze test that was performed to examine spatial cognition, the AD transgenic mice fed with traditional curcumin had no significant difference as compared with the vehicle control (Figure 4A). However, latency to the submerged platform and time spent significantly decreased at the end of the six sessions in the group fed with TML-6 (Figure 4A). Our behavior data therefore showed that the 3xTg AD transgenic mice fed with TML-6 showed significantly improved hippocampus-dependent learning behavior as compared with traditional curcumin (*p* = 0.0094) and the vehicle control (*p* = 0.0399, Figure 4A).

Following the behavior test, the animals were sacrificed and the mouse brain sections were examined by immunofluorescence staining for Aβ and Iba-1. The expression levels of Aβ in the brain of 3xTg AD transgenic mice fed with different chow diets are represented in Figure 4b. The data showed that the expression of Aβ was significantly reduced by 51% (*p* = 0.0035) in 3xTg AD mice fed with TML-6 as compared with the vehicle control (Figure 4B). However, 3xTg AD mice fed with traditional curcumin showed a trend of reduction but without significant differences (*p* = 0.1343), compared with the vehicle group (Figure 4B).

Brain inflammation is an important risk associated with AD progression. To investigate whether brain inflammation was also suppressed by TML-6, we examined the expression of Iba-1, a biomarker of microglial activation, in the groups of mice fed with vehicle, traditional curcumin, and TML-6. The data revealed that TML-6 significantly suppressed the expression of Iba-1 by 50% (*p* = 0.0473) in 3xTg AD mice in the brain region of hippocampus as compared with the vehicle control (Figure 4C). However, 3xTg AD mice fed with traditional curcumin had no significant change of Iba-1 expression levels as compared with the vehicle control (*p* = 0.6262) (Figure 4C).

In summary, TML-6 significantly improves the learning behavior of 3xTg AD transgenic mice. The expression of Aβ and Iba-1 levels in 3xTg AD transgenic mouse brains were reduced, which implies that hippocampus-dependent learning behavior was probably restored through the suppression of amyloid accumulation and brain inflammation by TML-6.

### 2.4. Pharmacokinetic Studies

**Figure 5:**
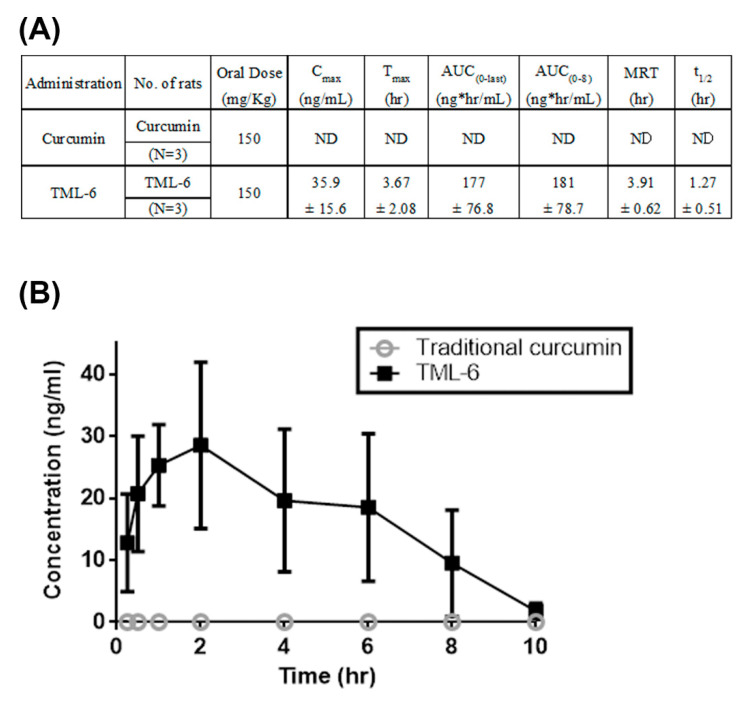
TML-6 exhibited significantly improved bioavailability as compared to traditional curcumin. (**A**) Summary of the absorption data on traditional curcumin and TML-6 in male Sprague–Dawley rats after oral administration of curcumin and TML-6 of 150 mg/kg. Values represented the mean ± SD of three rats in each treatment group. Abbreviations: ND, not detectable. (**B**) Bioavailability (serum concentration) of traditional curcumin and TML-6 was plotted against the time after oral administration to rats. The pK of curcumin was almost non-detectable (ND), consistent with the previous reports. Cmax indicates the observed maximum blood concentration and the time to reach the maximum blood concentration, while Tmax was determined directly from the experimental values. AUC (0-last) indicates the area under the concentration–time curve from time 0 to the last measurable concentration and MRT indicates the mean residence time.

According to previous reports, curcumin is difficult to be developed as a drug due to the poor bioavailability. Thus, the bioavailability of TML-6 is critical to be developed as a drug against AD. The pharmacokinetic (pK) study of curcumin and TML-6 was performed in blood after oral administration of 150 mg/kg in SD rats. The pK studies showed that the concentrations of curcumin at each time point were not detectable in rat blood, which is consistent with the poor bioavailability previously reported for curcumin (Figure 5A). However, the blood levels of TML-6 in plasma reached the peak levels of C-max of 35.9 ± 15.6 ng/mL and T-max of 3.67 ± 2.08 h (Figure 5A,B). This result indicates that the bioavailability of TML-6 shows a significant improvement as compared to traditional curcumin in SD rats after oral administration of 150 mg/kg curcumin and TML-6.

## 3. Discussion

In this study, we reported for the first time that the novel curcumin analog TML-6 may represent a drug candidate for AD therapy, meeting the current strategy to develop AD drugs targeting at multiple pathways in AD pathogenesis. TML-6 exhibited multiple biological effects on combining aging biology and AD pathogenesis, including the inhibition of APP and Aβ synthesis, the upregulation of ApoE, the transcriptional activation of the anti-oxidative *Nrf2* gene, the suppression of phospho-mTOR, and the inhibition of proinflammatory phospho-NF-κB. Importantly, the 3x-Tg AD mouse studies revealed improvements in learning behavior, reduced accumulation of Aβ levels, and suppression of the microglia activation marker Iba-1 in the brain. Although similar to the biologic effects of traditional curcumin, TML-6 exhibited significantly potent biological activities of up to 5–10-fold at each dose concentration as compared to curcumin. Furthermore, TML-6 showed considerably improved pharmacokinetic levels in the plasma as compared to the almost undetectable levels for traditional curcumin.

The potential of curcumin for clinical therapy has been a long-term huge controversy [43]. This controversy results from its pan assay interference compound (PAIN) [33,34] and poor bioavailability [44]. Both issues are the major medical concerns to be solved by chemical interaction studies, the formulation optimization, and clinical trial in the future drug development. In the past decades, hundreds to thousands of publications have reported on the in vitro and in vivo biological effects of curcumin in anti-aging and human diseases [36,45]. In order to mitigate this drawback of curcumin in drug development, many efforts have been made in the past decades to develop a bioavailable curcumin through drug delivery and formulation [44]. Recently, a few clinical studies using formulated curcumin have revealed significantly improved bioavailability and efficacy in human disease studies [46]. The most notable progress is the recent randomized, placebo-controlled trial report from UCLA which used a bioavailable form of curcumin for the therapy of non-demented AD adults. The results showed improved memory and reduced accumulation of Aβ/tau in the brain region by using 2-(1-{6-[(2-[F-18]fluoroethyl)(methyl) amino]-2-naphthyl} ethylidene)malononitrile (FDDNP) microimaging positron emission tomography (PET) after treating for 18 months [36,47]. This UCLA report offers the first proof of concept in therapeutic potential of formulated curcumin and its analog for AD therapy.

In agreement with previous reports, we demonstrated that curcumin and TML-6 can suppress the synthesis of APP [48]. Curcumin significantly decreased both Aβ40 and Aβ42 levels in rat neuroblastoma B104-APP751 and human neuroglioma H4-APP751 stable cell lines at 15 and 20 μM with no cytotoxicity. The inhibition of APP by curcumin was previously reported to mediate through the inhibition of the maturation process of the APP molecule. An interesting finding in this study is the remarkable inhibition of Aβ40 and Aβ42 synthesis by TML-6, but not by curcumin. Despite the cytotoxicity of the N2a/APPsw cell line found in the highest concentration of curcumin and TML-6, the Aβ40 and Aβ42 levels were significantly inhibited by TML-6 at 3.14 μg/mL, implying that the secretion of Aβ was reduced in the APP Swedish mutation-overexpressed N2a cell line (N2a/APPsw). This in vitro data were further verified in the AD transgenic mouse model, which showed significant reduction in Aβ in the mouse brain regions after feeding with TML-6. The current theory of AD pathogenesis is principally based on the study of early-onset AD patients, which showed alteration of mutant genes, such as APP, tau and presenilin [4,49]. Although the pathway involving APP/Aβ/tau represents the dominant mechanism of AD pathogenesis, the failure of many drug development efforts at the late stage trial raises the possibility that other non-amyloid AD mechanisms or alternative pathways should be explored in the development of therapeutics for AD [2]. In this scenario, our molecule TML-6, which exhibited multifaceted biological effects by combining the Aβ cascade and aging-related pathways, should shed light on the development of novel therapeutics for AD.

One new finding in this study is the demonstration of the upregulation of ApoE by curcumin and TML-6 for the first time. Apo E plays an important role in the lipid metabolism and the catabolism of Aβ clearance, which is closely related to AD pathogenesis [13]. Although several pathways associated with ApoE-related AD have been reported, the mechanism of ApoE in AD pathogenesis is not well elucidated. Cramer and colleagues reported that treatment with the retinoid X receptor (RXR) agonist bexarotene in AD transgenic mice resulted in the reversal of AD deficit, which is ApoE-dependent [13]. Preliminary studies in our laboratory revealed that curcumin and TML-6 upregulated ApoE expression through the PPARα/RXRα responsive elements in the ApoE promoter region. The ApoE levels in cerebrospinal fluid and plasma tend to be lower in patients with AD than in healthy individuals, as observed in cohort and meta-analysis studies [15,16]. Therefore, increasing the expression of Apo E in all *APOE* genotypes may provide a therapeutic strategy against AD.

One provocative theory in AD pathogenesis is the role of mTOR and the autophagy machinery mechanism. Recently, Lambeth and colleagues reported that long-lived protein can spontaneously modify substrates and gradually disrupt the lysosomal protein degradation pathway [50]. Therefore, activation of the cellular autophagy lysosomal system regulated by mTOR may provide one alternate strategy for aggregated protein clearance. In this study, both curcumin and TML-6 can cause downregulation of the mTOR pathway through suppression of phospho-mTOR for about 50% as compared to the control. mTOR is a key regulator of lifespan and affects aging-related pathogenesis such as mRNA translation, regulation of autophagosome, and metabolism [51,52]. A recent study showed that mTOR activation can enhance Aβ production by upregulating β- and γ-secretase cleavage [53]. Furthermore, the activation of mTOR may downregulate Aβ clearance through the dysfunction of autophagy machinery, which may subsequently result in the accumulation of Aβ deposition and formation [54,55]. Therefore, the inhibition of mTOR by TML-6 may activate autophagy machinery, not only providing a therapeutic strategy for AD, but also ameliorating the aging process.

**Figure 6:**
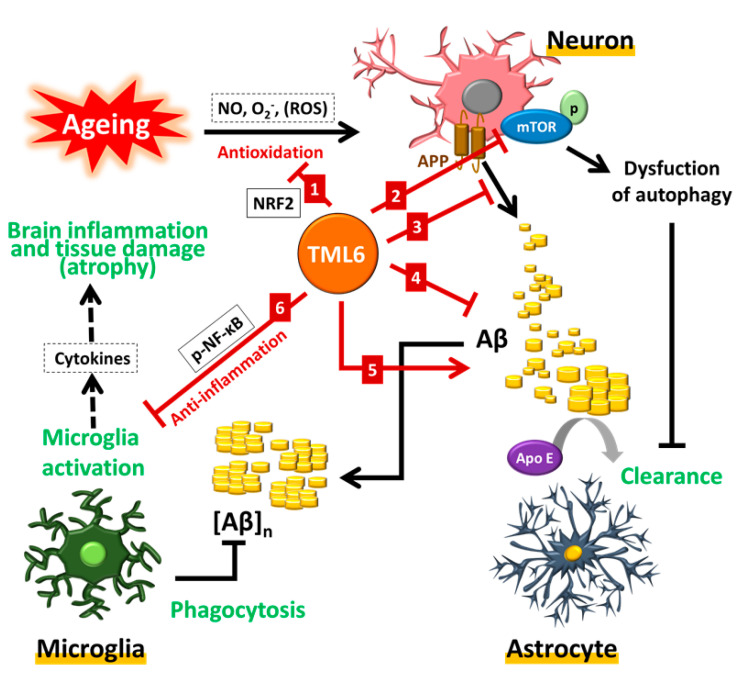
A hypothetical scheme on the potential action mechanism of TML-6 in the therapy of Alzheimer’s disease. Curcumin derivative-TML-6 treatments can induce (**1**) transcriptional activation of the Nrf2 promoter, which can reduce free radicals, the hallmark of the aging process; (**2**) following the aging process, the dysfunction of autophagy machinery and activation of mTOR will reduce the autophagolysosomal function to remove the misfolded proteins and lead to the accumulation of Aβ. TML-6 can effectively inhibit the phosphorylation of mTOR and maintain the normal function of autophagy to assure Aβ clearance; (**3**) TML6 can inhibit the production of Aβ from APP and (**4**) suppress the synthesis and secretion of Aβ from neurons; (**5**) TML6 can transcriptionally activate APOE and enhance the clearance of Aβ; (**6**) TML6 can inhibit the activation of microglial cells and reduce inflammatory injuries in the brain. The future design of therapeutics should combine targeting APP/the Aβ cascade and improving aging-related biological processes.

The most notable recent theory of AD pathogenesis is the unifying concept of the aging process as the driving role in neurodegenerative diseases and AD [56]. The emergence of free radicals has been reported to play a major role in the aging-related process [21]. Reactive oxygen species (ROS) are one kind of free radicals which may attack proteins, lipids, and DNA, leading to the activation of a series of mutagenesis processes in the aging process [57]. TML-6 could transcriptionally activate the *Nrf2* gene in a dose-dependent manner, with the highest activity at a concentration of 1.32 µg/mL. Currently, antioxidant and anti-inflammatory effects of the ketogenic diet can provide neuroprotection in AD through effective cognitive processes and support the concept of life style change in anti-aging and ameliorating AD progression [58]. The strong anti-oxidative effect of curcumin or TML-6 may initiate the downstream signal of aging or AD pathogenesis. Whether curcumin or TML-6 exhibits multiple biologic targets or, alternatively, they only exert an initiating anti-oxidative role of aging which then drives the subsequent aging biology, requires further clarification. These implications also indicate that the inhibition of upstream anti-oxidative ROS may explain the effects of TML-6 on the multiple downstream effects of TML-6, as shown in Figure 6. This possibility indicates the importance of the antioxidant effect on aging biology and AD, moving beyond the traditional amyloid and tau approaches.

Finally, a few challenges exist for future drug development of TML-6. One concern is the potential toxicity of TML-6 based on the IC50 of TML-6 in Huh-7, although our preliminary data on toxicological experiments showed no obvious toxicity in the rat model. The other challenges of TML-6 in preclinical studies will be to optimize the formulation development to improve the solubility and absorption of the unformulated TML-6 that was used in the cell biology and AD transgenic mouse model in this study. Preliminary data also showed a low brain penetration of 3 ng/mL (10% of the plasma pK levels) in AD mice receiving 150mg/kg of TML-6, while it was not detected in the brain of the normal mice receiving the same dose of it. These findings are unexpected but interesting considering the recent report on the dysfunction of BBB in the AD brain. In AD individuals, the deposit of Aβ in the vascular endothelium may result in angiopathy, thus, drugs or blood substance may gain access to neural tissues [59]. This possibility may explain the efficacy of TML-6 in improving the behavior and the reduction in Aβ in brain tissues that were observed in our AD transgenic mouse model.

## 4. Materials and Methods 

### 4.1. The Curcumin Analog TML-6

A panel of 12 synthetic curcumin analogs were obtained from the AndroScience Company (ASC, San Diego, CA, USA), the products of which were later acquired by the Allianz Biotechnology Company, Taipei, Taiwan. Due to the historical belief on the role of curcumin in anti-aging and AD prevention, Professor Ih-Jen Su of the Southern Taiwan University of Science and Technology started to screen these compounds by adopting 6 molecular markers related to aging and AD pathogenesis to identify a drug candidate for AD. The compound ASC-6, later re-named as TML-6, was finally selected as the candidate compound based on the data of cytotoxicity, biologic activities, and pharmacokinetics. The triplex-Tg AD mice were then conducted to evaluate the efficacy.

The active pharmaceutical ingredients of TML-6 were synthesized and physico-chemical properties were characterized by SinoPharm Pharmaceutical Company (SPT), Tainan, Taiwan. Preformulation studies on the solubility of TML-6 were conducted by Value Pharmaceutical Services (VPS) Company, Jiansu, China. All the data in this study were conducted using unformulated TML-6. Formulation to optimize the solubility and permeability of TML-6 is under development for toxicological studies.

### 4.2. Cell Lines, Cell Culture, and Chemicals

A total of 5 cell lines (Huh-7, HepG2, SH-SY5Y, U373MG, and N2a) were examined for the feasibility of expression of aging and AD-related proteins. Among them, two cell lines were chosen for the working model for screening curcumin derivatives based on the dose-dependent expression of APP and ApoE. The human hepatoma Huh-7 cell lines were obtained from the Health Science Research Resources Bank (JCRB0403; Osaka, Japan) and the mouse neuroblastoma cell line N2a cells with stable overexpression of full-length APP and APP Swedish mutations, designated as N2a/APPswe, were kindly provided by Prof. Kuo, Yu-Min, National Cheng Kung University Medical School, Tainan, Taiwan. For cell biology studies, TML-6 was dissolved in DMSO to obtain stock solution. Both cell lines were then cultured in Dulbecco’s modified Eagle’s medium (DMEM, Gibco, Grand Island, NY, USA) supplemented with 100 U/mL penicillin, 100 mg/mL streptomycin and 10% fetal bovine serum (FBS, Biological Industries, Cromwell, CT, USA). Cells were cultured at 37°C in a humidified atmosphere of a 5% CO_2_ incubator (Forma Scientific, Marietta, OH, USA). The cells were seeded in 24-well plates, or 6- or 10- cm dishes overnight on the day before the experiment. On the next day, the cells were treated with traditional curcumin (Sigma, St. Louis, MO, USA) and TML-6. The working concentration of each compound is indicated in the figures.

### 4.3. Cytotoxicity Assay

The cytotoxicity assay was performed using the Cell Counting Kit-8 (CCK-8, Sigma). We dissolved 0.0524 g of TML-6 (molecular weight is 523.62 g/mol) in 10 mL DMSO to obtain TML-6 stock at 10 mM for cell line study. The working concentrations at 0.3125, 0.625, 1.25, 2.5, 5, 10 and 20 μM were diluted from 10 mM TML-6 stock in culture medium. For data presentation in cytotoxicity assay, we converted the working concentration of μM into μg/mL as indicated in Figure 2A. Briefly, Huh-7 cells were seeded overnight in 96-well plates at 5000 cells/well and then subjected to traditional curcumin or TML-6 treatment for 24 h. At each time point, a CCK-8 solution (0.25 mg/mL) was added to the medium in each well for an additional 4 h incubation, and absorbance was measured at 570 nm using an ELISA reader.

### 4.4. Cell Lysis and Western Blotting

After cells were seeded and treated with curcumin and TML-6 for 24 h, cells were washed with ice-cold PBS (Phosphate Buffered Saline) and lysed with 100 μL of RIPA cell lysis buffer (Pierce Biotechnology, Rockford, IL, USA) supplemented with protease and phosphatase inhibitors (Roche Diagnostics, Mannheim, Germany). Cell lysates were further cleaned by centrifugation at 14,000 rpm at 4 °C for 15 min. Protein concentration was determined using a Bradford assay (Bio-Rad, Hercules, CA, USA). For Western blot analysis, 20 μg of each sample was prepared for SDS-PAGE (SDS Poly-acrylamide-gel-electrophoresis) and denatured at 95 ℃ for 10 min. The samples prepared from total lysates were loaded and separated by SDS-PAGE (8% or 10%), and then transferred to PVDF membranes (pore size 0.45 μm; PerkinElmer, Waltham, MA, USA) by semi-dry transfer cell (Bio-Rad). After blocking with 5% skim milk in PBS-T (0.1% Tween-20), the membranes were hybridized with primary antibodies at 1/1000 dilution at 4 °C overnight. On the next day, membranes were washed with PBS-T followed by incubation with HRP-conjugated anti-mouse or anti-rabbit secondary antibody (DAKO, Santa Clara, CA, USA) at 1/5000 dilution at room temperature for 1 h as appropriate. Finally, the images were captured by Amersham Imager 600 (GE Healthcare Life Sciences, Marlborough, MA, USA) after being developed using the chemiluminescent HRP substrate (Millipore, Bedford, MA, USA). The primary antibodies used in this study were anti-APP, anti-NF-κB (Cell Signaling Technology, Danvers, MA, USA), anti-Apo E, anti-p-mTOR (Abcam, Cambridge, MA, USA).

### 4.5. Amyloid β Enzyme-Linked Immunosorbent Assay

Extracellular levels of soluble Aβ40 and Aβ42 were determined by LEGEND MAX™ β-Amyloid x-40 and X-42 ELISA Kit (Biolegend, San Diego, CA, USA). Overexpression of the APP Swedish mutation (APPswe) in the N2a neuronblastoma cell line was called the N2a/APPswe cell line. N2a/APPswe stable cells were seeded at 8 × 10^5^ cells in 6 cm dishes. After overnight incubation, the cells were treated with curcumin and TML-6 for 24 h. The working concentrations of curcumin and TML-6, as indicated in Figure 2, were prepared by dissolving the TML-6 in DMSO as stock solution and then diluted in 3 mL DMEM medium. The culture supernatant was collected and centrifuged at 1500× g at 4 °C for 5 min to remove cells. The culture supernatants from various treatments were conducted to examine the Aβ levels according to the manufacturer’s instructions. The samples for the detection of soluble Aβ levels were diluted in the supernatant at a 1:10 dilution for this experiment. Levels of soluble Aβ were detected by measuring the OD at 620 nm (Molecular Devices, San Jose, CA, USA). The calculation of Aβ levels was based on the standard curves of Aβ40 and Aβ42, respectively.

### 4.6. Luciferase Reporter Assay

To examine the activities of the anti-oxidative gene NrF2, Huh-7 cells were seeded in 24-well culture plates and then co-transfected with serial dilution of pG5-Nrf2-Luc (firefly luciferase) with the pRL-TK Renilla luciferase reporter. Following overnight incubation, the transfected cells were treated with traditional curcumin and TML-6 compounds for additional 24 h. The luciferase reporter assay was performed using the Dual-Luciferase Reporter assay system (Promega, Madison, WI, USA). The cell lysates were assayed for both firefly and Renilla luciferase activities. The relative luciferase units (RLU) were measured using a luminometer (GLOMAX Multi, Promega). Firefly luciferase activity was normalized for transfection efficiency using the Renilla luciferase activity in each lysate as the control. The RLUs are expressed as means plus standard deviation of three independent experiments.

### 4.7. AD Transgenic Mice and Diets

The triplex transgenic mouse model (3xTg-AD, APP Swedish, MAPT P301L, and PSEN1 M146V) was adopted as the AD model and bred at the laboratory animal center of National Cheng Kung University (NCKU), Tainan, Taiwan [60]. The mice were housed in a room maintained on a 12 h light–dark cycle and fed ad libitum. Six-month old 3xTg-AD mice were used for animal experiments. Experimental procedures for handling the mice were in accordance with the guidelines of the Institutional Animal Care and Use Committee (IACUC) of NCKU. These mice develop plaques and tangles pathology. Aβ deposition is progressive, and deposition appears as early as nine months for triplex-Tg mice. The AD transgenic mice were fed with the control diet (Laboratory Rodent Diet 5001, LabDiet, St. Louis, MO, USA), the control diet plus traditional curcumin (150 mg/kg), or the control diet plus TML-6 (150 mg/kg) for four months. Diets were stored at 4 °C and were added to mouse cage twice a week. The quantity of the diet consumed by the mice was determined daily. The concentration of curcumin and TML-6 in the diet was determined to be around 1.20 mg/g of diet after assaying by HPLC by the Development Center for the Biotechnology (DCB), Taipei, Taiwan.

### 4.8. Morris Water Maze Test

For assessing spatial learning and memory, the Morris water maze was used as we described before [61,62]. Mice were trained to memorize the location of a hidden platform in a swimming pool (120 cm diameter, 50 cm depth). The platform was 1 cm below the water surface. There were four different cues in each quadrant. Mice were allowed to explore the maze for four trials per session and 6 sessions in total in 6 days. Each trail lasted for 120 s and the resting time was 30 s. The time taken to reach the platform was recorded as escape latency.

### 4.9. Immunofluorescence Staining

Mice were anesthetized using pentobarbital (60 mg/kg, intravenous) and then transcardial perfusion was performed with 0.01 M PBS (pH 7.4). The brains were post-fixed in 4% PFA (paraformaldehyde) for 2 days and dehydrated with 30% sucrose at 4 °C. The hydrated brain was embedded with optimal cutting temperature compound (OCT, Leica, Wetzlar, Germany) and quick-freeze was performed at −30 °C. The blocks were sectioned into 16 µm-thick slices. Slices were stored at −20 °C. Before immunofluorescence staining, the sections were soaked in cold PBS for 5 min. Antigen retrieval was performed with 0.01 M citric acid at 100 °C for 5 min. The sections were then blocked with 5% normal donkey serum (Millipore) containing 0.1% Triton X-100 (Sigma) in PBS-T. Primary antibody Iba1 (Wako, Richmond, VA, USA, 1:400) and Aβ (Covance, Princeton, NJ, USA, 1:100) were used at room temperature for 1 h. Alexa Fluor 488-conjugated anti-Rabbit antibody (for Iba1, Invitrogen Life Technologies, Grand Island, NY, USA, 1:300) and Alexa Fluor 488-conjugated anti-mouse antibody (for Aβ, Invitrogen Life Technologies, 1:300) were used, respectively. Images were captured by the TissureFAXS microscopy system and positive signals were quantified by TissueQuest software module (TissueGnostic, Vienne, Austria).

### 4.10. Pharmacokinetic Studies

In order to evaluate the bioavailability of curcumin and its analog TML-6, pharmacokinetic studies were commissioned and conducted by the Development Center for Biotechnology (DCB, Taipei, Taiwan). The kinetics of curcumin and TML-6 in blood were collected at 0.25, 0.5, 1, 2, 4, 6, 8 and 10 h after oral administration of curcumin and TML-6 at the dose of 150 mg/kg in male Sprague–Dawley Rats. 

### 4.11. Statistical Analysis

Significant differences between traditional curcumin and TML-6 in the Nrf2 promoter (assessed by the luciferase reporter assay), Aβ production in the N2a/APPswe stable cell line, and expression levels of Aβ and Iba-1 in transgenic AD mice were determined by paired *t*-test * *p* < 0.05, ** *p* < 0.01, *** *p* < 0.001. Data represent the mean with a standard deviation (SD) error bar.

## 5. Conclusions

Together with many other reports in the literature on AD pathogenesis, we propose a work model to unify the complex pathogenesis of AD into one scenario based on the aging biology as described in Figure 6. The increasing prevalence of sporadic AD in the elderly suggests that the early events of AD may start with the aging process in the fifth decade of age in at-risk individuals. With the evolution of the aging process, oxidative stress prevails, which is followed by the dysfunction of the autophagy machinery, manipulating the misfolded proteins in the neurons of the aged individuals. The reduction in ApoE may not only worsen the cholesterol metabolism but also exacerbate amyloid plaque formation, which will trigger microglial activation and inflammation, leading to neuronal loss and brain atrophy. In agreement with the current concept to treat early-stage or non-demented AD patients, the gold standard for future drug development for AD should not only reduce Aβ accumulation but must also improve the pathobiology associated with the aging process. In this scenario, the curcumin analog TML-6 meets the current strategy to develop therapeutics for the prevention and therapy of AD.

## Abbreviations

3xTg	triple-transgenic
Aβ	amyloid-beta
AD	Alzheimer’s disease
APP	amyloid precursor protein
Apo E	apo-lipoprotein E
BBB	blood-brain barrier
CCK-8	cell Counting Kit-8
DMEM	Dulbecco’s modified Eagle’s medium
DMSO	dimethyl sulfoxide
DNA	deoxyribonucleic acid
ELISA	enzyme-linked immunosorbent assay
FBS	fetal bovine serum
HRP	Horseradish peroxidase
Iba-1	ionized calcium-binding adapter molecule 1
IC50	half maximal inhibitory concentration
mTOR	mammalian target of rapamycin
ND	not detectable
NF-κB	nuclear factor kappa-light-chain-enhancer of activated B cells
Nrf2	nuclear factor erythroid 2-related factor 2
PBS	phosphate-buffered saline
PFA	paraformaldehyde
pK	pharmacokinetic
ROS	reactive oxygen species
SDS-PAGE	sodium dodecyl sulfate polyacrylamide gel electrophoresis
SD	standard deviation
